# Analysis of the Dynamic Proteasome Structure by Cross-Linking Mass Spectrometry

**DOI:** 10.3390/biom11040505

**Published:** 2021-03-27

**Authors:** Marta L. Mendes, Gunnar Dittmar

**Affiliations:** Proteomics of Cellular Signaling, Department of Infection and Immunity, Luxembourg Institute of Health, 1a Rue Thomas Edison, 1445 Strassen, Luxembourg; marta.mendes@lih.lu

**Keywords:** proteasome, cross-linking mass spectrometry, structural biology, X-ray crystallography, electron microscopy

## Abstract

The 26S proteasome is a macromolecular complex that degrades proteins maintaining cell homeostasis; thus, determining its structure is a priority to understand its function. Although the 20S proteasome’s structure has been known for some years, the highly dynamic nature of the 19S regulatory particle has presented a challenge to structural biologists. Advances in cryo-electron microscopy (cryo-EM) made it possible to determine the structure of the 19S regulatory particle and showed at least seven different conformational states of the proteasome. However, there are still many questions to be answered. Cross-linking mass spectrometry (CLMS) is now routinely used in integrative structural biology studies, and it promises to take integrative structural biology to the next level, answering some of these questions.

## 1. A Macromolecular Degradation Machine

The proteasome is the most important protease for regulated proteolysis and regulates many cellular processes, including cell cycle control, regulation of inflammation, and switching metabolic processes. As a central regulator, the proteasome has been a target for drug development and is important for treating several cancers. 

**Figure 1:**
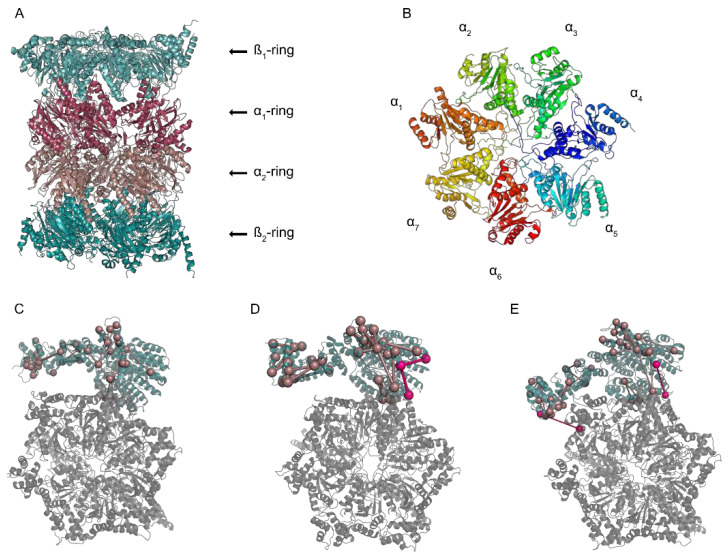
Structure of the 20S proteasome and its interaction with the 19S. (**A**) Side view of the 20S proteasome showing the four rings (PDB: 5WVI). (**B**) Top view of the 20S proteasome with the seven subunits shown in different colors (PDB: 5WVI). (**C–E**) Crosslinks identified by cross-linking mass spectrometry (CLMS) between the AAA-ATPase heterohexameric ring and the RPN1 subunit of the 19S regulatory particle (PDB: 4CR2), adapted from Mendes et al 2019 [6].

The proteasome is a 2.5 MDa macromolecular machine that degrades proteins in an ATP-dependent manner. Its core is the 20S core particle, a barrel-shaped complex, which harbors the different proteolytic sites in its inner channel (Figure 1). Although generally seen as a latent protease, the 20S can degrade some proteins, as described in Sahu and Glickman’s review in this Special Issue [1]. The 20S proteasome can associate with different activator complexes (19S, PA200, and PA28), forming a larger complex. Among the activators, the 19S complex is the most common one, which leads to the formation of the 26S (single-cap) or 30S (double-cap) complexes. More details on the structure and functions of PA200 and PA28 are provided by two articles in this Special Issue [2,3]. The 26S proteasome is the endpoint for proteins marked by the ubiquitin enzyme cascade for degradation and leads to their destruction into peptides and amino acids [4,5].

## 2. The Pieces of the Puzzle

The 26S proteasome is composed of at least 33 subunits, divided into two main complexes, the 20S core particle (CP) and the 19S regulatory particle (RP). The 20S proteasome is composed of four heteroheptameric rings, each formed by seven subunits, either α- or β- types, that lay on top of each other forming a cylinder-shaped barrel in an α1-7β1-7β1-7α1-7 very stable arrangement (Figure 1A,B) [7,8]. The 19S proteasome can be separated into the lid and the base [9]. The lid comprises nine regulatory particle non-ATPase subunits: RPN3, RPN5, RPN6, RPN7, RPN8, RPN9, RPN11, RPN12, and RPN15, and the base is composed of the regulatory particle ATPase subunits (RPT1–6) forming a heterohexameric ring and the subunits RPN1, RPN2, RPN10, and RPN13 (reviewed in [10]). The first step of the proteasome degradation process starts with recognizing ubiquitinated substrates by the ubiquitin receptors RPN1, RPN10, and RPN13 in the base of the 19S proteasome [11,12,13]. Ubiquitin chains are then cleaved by the deubiquitinase (DUB) RPN11 in the lid [14,15], and the substrate is translocated to the AAA+ ATPase heterohexameric ring where it is unfolded and translocated to the 20S proteasome for degradation ([16], reviewed in [17]). Apart from the 33 subunits in the proteasome, several extrinsic proteins are also implicated in the degradation process. Ubiquitinated targets can be recognized and recruited to the proteasome through extrinsic ubiquitin receptors, such as RAD23 and DSK2 [18,19]. USP14 and UCH-L5 work as two additional stable associated DUBs [20,21]. The 26S proteasome is a highly dynamic structure that goes through at least seven conformational states while recruiting the target and its degradation.

## 3. Solving the Structure of the Proteasome

To this date, around 400 structures of the proteasome (20S and/or 19S—advanced search query for *Proteasome* as structure title.) have been deposited in the Protein Databank (https://www.rcsb.org). Although these structures contributed enormously to understanding the function of the proteasome, due to its highly dynamic nature, a lot is still to be discovered until we fully understand this molecular degradation machine. 

X-ray crystallography is a well-established, high-resolution technique that can be applied to proteins and complexes of almost all sizes and complexities to solve their structures [22]. However, these proteins and complexes must crystallize, and in many cases, like in the case of membrane proteins and large or labile complexes, this is a highly complex task [22]. Due to its stability, the 20S proteasome was the first complex of the 26S proteasome to have its structure solved by X-ray crystallography. Two landmark papers delivered the first structures for the *Thermoplasma acidophilum* and *Saccharomyces cerevisiae* 20S proteasome [7,8]. Both structures were in the resting state and, in the year 2000, the first open gate structure was delivered by the same technique [23]. It was not until the mid-2010s that the first structure of the human 20S proteasome, in the resting state, was also solved by X-ray crystallography [24,25]. X-ray crystallography was used to solve the structures of some proteins of the 19S proteasome activator; however, due to its highly dynamic nature, the entire 19S proteasome complex could not be solved.

Cryo-electron microscopy (cryo-EM) came up as an alternative to X-ray crystallography. Although very expensive and until recently low resolution, it can be applied to all types of samples in a near-native state [22,26], and it has been the technique of choice to study the structure of the 19S proteasome activator. In the late 2000s, two groups reported low-resolution structures of the 26S proteasome from Drosophila and Schizosaccharomyces pombe, respectively [27,28]. In the mid-2010s, with advances in hardware and software, higher resolution structures could be obtained by cryo-EM. In 2014, several co-existing conformational states in the *S. cerevisiae* proteasome were reported [29], and today, seven co-existing states of the human proteasome are known [30,31,32].

## 4. The New Runner in the Race for Dynamic Structures—Cross-Linking Mass Spectrometry

The highly dynamic structure of the 19S proteasome and the many conformational states that it goes through during protein degradation have prevented both X-ray crystallography and cryo-EM from completely revealing the proteasome assembly, leaving several questions still to be answered. Cross-linking mass spectrometry (CLMS) has become a well-established methodology that, used in an integrative manner, can provide useful information on proteasome dynamics.

Cross-linking was first coupled to mass spectrometry in the beginning of the 1990s. Examples are the use of CLMS to determine the multimeric state of proteins and to identify the interfacing domains in recombinant human erythropoietin [33,34]. After years of instrumentation, method, and software development, CLMS is now a well-established methodology in many laboratories. 

**Figure 2:**
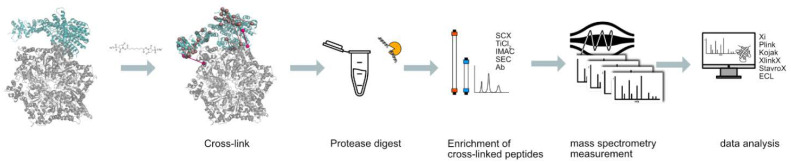
Workflow of a CLMS analysis. The protein or protein complex of interest is cross-linked, followed by protease digestion to generate peptides suitable for mass spectrometric analysis. This is followed by an enrichment step for crosslinked peptides, which are in turn sequenced by mass spectrometry. The resulting spectra are interpreted by specialized software packaged to reveal the location of the cross-linked peptides on the protein/complex structure.

Several workflows have been published in order to try to increase the yields of identified cross-linked peptides, but they all converge in five main steps [35,36,37]: the cross-linking of the protein, or proteins, of interest with the chosen cross-linker; the digestion of the cross-linked proteins by a protease, or proteases; the enrichment of cross-linked peptides; the analysis of cross-linked peptides by liquid chromatography–coupled mass spectrometry (LC-MS/MS); and the identification of the cross-linked peptides (Figure 2).

Various types of cross-linkers, with different reactive groups and spacer arm lengths, are available to be used in CLMS workflows, and their choice is important depending on the application [38]. For instance, shorter cross-linkers will be more suitable for structure prediction [39]. Examples of the most used cross-linkers are DSS/BS3, which have the same reactive groups at both ends reacting with primary amines; SDA, which has different reactive groups at both ends reacting with primary amines on one end and any amino acid side chain on the other, thus generating a higher combination of cross-linked peptides [40]; and EDC, a zero length crosslinker that reacts with primary amines on one end and carboxyl groups on the other end. One of the major issues of cross-linked peptide identification is that during MS analysis, one of the peptides always fragments better than the other originating complicated spectra, thus hindering identification [41]. Cleavable cross-linkers, like DSSO, which are fragmented during MS acquisition, were developed to address that problem [42]. 

Enrichment of cross-linked peptides is an essential step in CLMS due to the normally low levels of cross-linked peptides obtained. Enrichable cross-linkers are among the enrichment options. These cross-linkers, besides the two reactive ends, contain a tag that can then be used to enrich the cross-linked peptides. Although biotin is probably the most common tag used in this type of cross-linker [43,44], there are other types of enrichable cross-linkers. PhoX and pBVS have a phosphate-based tag, which can be enriched by immobilized metal affinity chromatography (IMAC) and titanium dioxide (TiO_2_), respectively [45,46]. Cross-linked peptides consist of two linear peptides joined by a cross-linker; they are thus higher charged and larger than linear peptides. Strong cation exchange chromatography (SCX) and size exclusion chromatography (SEC) can be used, either separately or together, to enrich cross-linked peptides, by charge and/or size, respectively [47]. 

The identification of cross-linked peptides is also a challenge. Instead of one linear peptide, there are now two peptides connected by a cross-linker, and all the possible combinations of peptide pairs must be considered, increasing the search space quadratically. Depending on the size of the database, searches can be very time consuming and the risk of false positive identifications can increase. Several software packages, such as Xi [6], Plink [48], Kojak [49], XlinkX [50], StavroX [51], and ECL [52], among others [36,53], have emerged to identify cross-linked peptides (Figure 2).

Despite the challenges, there are major advantages in using CLMS: it can be performed in the native state, in solution, with low sample amounts, and with no need for sample purification. Although CLMS cannot deliver high-resolution structures, it can give valuable information on the dynamics of protein complexes. Therefore, it is commonly used alone or in combination with other structural biology techniques in integrative studies. Examples for the use of CLMS are the determination of the relative positions of ATPases to the alpha-type subunits of the 20S proteasome or the arrangement of the AAA-ATPase ring in fully assembled proteasomes [54,55]. Combining EM with CLMS allowed the definition of an atomic model of the AAA-ATPase-CP sub-complex [56]; combining cryo-EM and CLMS clarified the topology of the AAA-ATPase complex and its positioning relative to the alpha-ring of the 20S proteasome, leading to a model for the early steps of protein degradation by the 26S proteasome [27]. Insights into the molecular architecture of the 26S proteasome obtained by X-ray crystallography, cryo-EM, and CLMS shed light into the sequence of events before degradation of ubiquitinated substrates [57]. More recently, using cryo-EM and CLMS, two studies showed how the biogenesis of the lid triggers the lid and base assembly [58,59]. Another study, using quantitative CLMS, on the influence of oxidative stress revealed an influence on proteasome dynamics. Here, the interface between the 20S and the 19S proteasome seems to rotate under these conditions [60]. Mendes et al. used CLMS to show the coexistence of four different conformational states of the proteasome [6] (Figure 1C–F).

## 5. Where Are We Heading?

Despite all of the efforts to understand the dynamics and function of the proteasome, a lot is still to be discovered. Are there more states of the proteasome? How does the proteasome structure change during ubiquitin chain recognition? These are some of the multiple questions still to be answered. 

CLMS has shown its potential as a structural biology integrative technique, and although it has been widely used to study purified protein complexes, advances in the field made it possible to successfully cross-link whole-cell lysates with yields in the range of the 10,000 cross-linked peptides [61,62]. O’Reilly et al. have shown that CLMS can be used as an integrative in-cell structural biology method by delivering the architecture of the expressome in *Mycoplasma pneumoniae* in its native state [62]. As for the proteasome, being such a highly dynamic machinery, an integrative in-cell structural biology strategy, including the more established methodologies in structural biology and CLMS, should give more insights into the structure and function of the proteasome. Recent advances in the mass spectrometry field, such as the ion mobility cells that are now improved and more accessible to researchers in the mass spectrometry field, should also improve detection and identification of cross-linked peptides [63]. If that promise holds true, the power of CLMS as an integrative structural biology tool will be even higher and will soon offer more answers on the structure and functionality of the proteasome.

## Data Availability

Not applicable.
